# Out with the old, in with the new? Institutional experimentation and decent work in the UK

**DOI:** 10.1177/0143831X231220528

**Published:** 2024-01-19

**Authors:** Mathew Johnson, Eva Herman

**Affiliations:** Work and Equalities Institute, Alliance Manchester Business School, UK

**Keywords:** De-regulation of industrial relations, industrial democracy, institutional change, mutual gains, union organising

## Abstract

Drawing on an extended critical case study of the Greater Manchester (GM) city region in the UK, this article contributes to debates around the changing role of social actors within local labour markets, and how they contribute to processes of regulatory experimentation and innovation. While recent literature has drawn attention to new actors and novel strategies in responding to labour market disruptions, in this article the authors argue that there is still room for embedded actors and established practices in defending, and advancing, decent minimum standards. This may be through political lobbying, workplace organising, industrial action, extending collectively agreed standards to outsourced workers, or through hybrid forms of trade union–community campaigning. Against a wider background of labour market de-regulation, the authors’ case study points to the layering up of increasingly fluid and context-specific repertoires of conflict and cooperation that shape labour market ‘norms’ and legitimise particular progressive causes within local rather than national capitalisms.

## Introduction

Within the fields of comparative industrial relations and political economy, a growing body of literature has explored the efforts of myriad social actors to contest management behaviours that fissure workplaces, erode social welfare, and disrupt agreements over wages, contract types and working practices (e.g. Murray et al., 2020; O’Brady, 2021). Focusing on the interplay between structural and agentic power, some note that trade unions have been forced to experiment and innovate in response to the pressures of marketisation, regulatory fragmentation and employer opportunism (e.g. Benassi and Vlandas, 2016; Però, 2020), but these seemingly ‘tactical’ responses may also create the solidaristic foundations for broader processes of re-regulation and institution building across the labour market (Doellgast et al., 2018; López-Andreu, 2020). ‘New’ actors in the labour market such as indie unions and alt-labour groups are seen to deploy ‘new’ repertoires of contention such as direct action against employers and state actors, organising consumer boycotts and generating campaign pressure through social media (e.g. Heery, 2018; Milkman, 2019; Però, 2020). Civil society organisations (CSOs) may also fill some of the gaps left by retreating state services (e.g. Griffin, 2023; Mustchin et al., 2023) and can (partly) compensate for the weak enforcement of existing rules by regulating labour markets ‘from below’ (Fine and Bartley, 2019; Lesniewski and Canon, 2016). But as this article will argue however, there is still room for ‘old’ actors and ‘embedded’ practices in regulating the labour market. The state still directly and indirectly shapes standards through a combination of hard and soft law (Cunningham et al., 2022; MacKenzie and Martínez Lucio, 2014; Yates and Clark, 2021), and collective bargaining agreements continue to be important reference points for pay and conditions (Brandl, 2022). Traditional unions increasingly recognise the need to reach out to precarious workers (Doellgast et al., 2018; Smith, 2022) and can provide the institutional resources needed to sustain social justice campaigns and coalitions (e.g. Rhomberg, 2018; Tattersall, 2018).

Drawing on a longitudinal case study of the Greater Manchester city region in the UK (2020–2023), we map the various actors, organisations and social reference points that, in combination, seek to regulate local labour markets in the face of increasingly globalised pressures on labour rights and standards. Our findings suggest that, on the one hand, a broadly stable and progressive local political context in Greater Manchester has legitimised ‘partnership’ working between the state, employers and trade union leaders to set decent minimum standards in the labour market. On the other hand, an increasingly diverse and assertive labour movement and civil society are able to draw on their considerable (but somewhat fragmented) structural and discursive power to push back against disruption and work degradation.

Positioning the specific case of Greater Manchester within a wider context of ‘neoliberal disruptions’ (Murray et al., 2020) allows us to make two substantive contributions. The first is to show that rather than simple dichotomies of new vs old practices, and experimentation vs defending the status quo, what we increasingly see is the ‘layering up’ of multiple re-regulatory strategies as innovations interact with and build on embedded practices, giving rise to increasingly fluid and contingent repertoires of conflict and cooperation (Castellani and Roca, 2022; Thelen, 2009). We argue that while novel approaches can be important as existing structures and strategies lose their power and relevance, they rarely appear overnight; rather they may emerge in incremental and path-dependent ways, drawing on the successes and failures of earlier experiments, and the legacies and networks of embedded social actors. The second contribution is to the international and comparative literatures on labour market restructuring and underlines the importance of understanding both context and scale in relation to the nature of disruption and the range of countervailing strategies deployed by social actors. The findings from our case study of Greater Manchester point to the growing importance of progressive city politics that do not map neatly on to existing national typologies (Jacobs et al., 2021; Joy and Vogel, 2021). But while top-down experiments (e.g. led by politicians and established social actors) are important to extend and legitimise decent minimum standards to precarious workers, this approach in the UK often relies on soft power and persuasion rather than enforceable ‘hard’ law. Conversely, bottom-up regulation has emerged somewhat more slowly and unevenly in the UK when compared with the US, and relies heavily on the resources, skills and network power of embedded activists. We argue that an ongoing dialogue between these different spheres of influence framed by the specific contextual dynamics and traditions of local rather than national capitalisms (Gough, 2014; O’Brady, 2021) is critical for understanding how labour market norms evolve over time.

The article is organised as follows. The first section reviews contemporary literature on the emergence of organisational and institutional experimentation in response to labour market disruptions. It then briefly considers the interaction of experimentation, embedded practices and hybridisation, and the emergent role of legitimacy in shaping labour local market norms. The third section sets out the qualitative research design and methods (oriented at the sub-national level), followed by the empirical data that are drawn from an in-depth case study of the Greater Manchester city region. We then discuss the findings and offer some conclusions and potential lessons around the changing nature of regulation in local labour markets.

## Labour market disruption and the decline of traditional institutions

Murray et al. (2020) identify a number of ‘fault lines’ that have disrupted traditional modes of work regulation and pose an existential threat to the broadly solidaristic foundations of the economic and social model that developed in many countries during the post-war era. ‘Neoliberal disruptions’ include growing global competitive pressures and the rise of financialisation, the spread of new technologies and modes of production, the unbundling of firms and vertically integrated supply chains through offshoring and outsourcing, and the marketisation and fragmentation of public services. All of these may all act as potential ‘ruptures’ in the institutional framework, leading to an erosion of the key features of the standard employment relationship (SER) that were hard won through collective bargaining, namely: secure work, a liveable wage, access to social protection and opportunities to participate (Bosch, 2004). While the SER was by no means universal even under corporatist systems of labour relations with a strong social wage, in many European countries there have been clear trends towards the ‘re-commodification’ of labour since the 1980s leading to widening inequality and the ‘normalisation’ of precarious work (Rubery et al., 2018).

**Figure 1.**
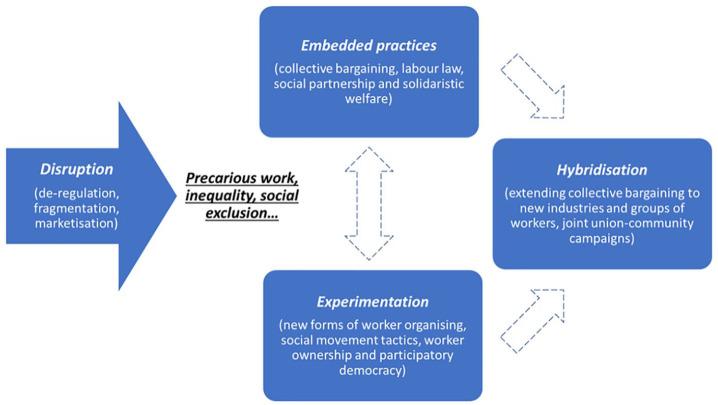
A schematic diagram of the varied responses to disruption.

These disruptions pose significant challenges to both the institutional foundations and legitimacy of existing systems of labour market regulation (such as they are), and ‘invite’ responses from social actors that help develop collective capabilities in order to resist downward pressure on decent minimum standards. This may entail the mobilisation of embedded power resources and practices to both defend and advance existing rules and frameworks, the deployment of new practices and power resources through experimentation in order to re-regulate work and employment, or hybrid solutions that combine elements of embedded and new practices (Figure 1).

### Embedded practices

An extensive body of literature has explored how trade unions, in the face of sustained pressure since the 1980s, have sought to consolidate membership density and collective bargaining coverage in core industries such as manufacturing, transportation and public services (e.g. Doellgast et al., 2018; Pulignano et al., 2015). Trade unions may leverage their institutional power resources to defend the interests of members through established mechanisms of collective representation, political lobbying and recognised legal procedures, and have organised and mobilised members within workplaces to challenge inequality and contest management opportunism (e.g. Benassi and Vlandas, 2016). The challenge here is that the steady decline of collective bargaining coverage in many countries, coupled with increasing segmentation between sectors, has left large shares of workers, and particularly those in precarious jobs, beyond the scope of existing institutional protections (Grimshaw et al., 2016). While some have argued that unions have neglected those in precarious and marginal jobs such as younger workers, women and migrants (Rueda, 2006), this may be an unintended outcome of a defensive survival strategy against the disruptive forces of contemporary capitalism (Murray et al., 2020) rather than *a priori* insider–outsider strategising. As Pulignano et al. (2015: 821) argue, dualism is likely ‘not a first choice but, at most, a second-best option for unions that do not have the strength . . . to protect all workers’. Indeed, the weakening of labour market institutions has been exacerbated by the shrinking generosity and coverage of social protection, and labour law that is increasingly out of step with new business models giving rise to significant protective gaps (Grimshaw et al., 2016). The increasingly perforated nature of industrial relations systems provides significant scope, and arguably the necessity, for actors to experiment with new practices.

### Experimentation

In this context, it is clear that experimentation may be necessary in order to both defend, and advance, the interests of workers, and to contest disruption. These experiments may be top-down in nature and driven by established actors in the labour market such as politicians and policy makers, or they may be more organic and ‘bottom-up’ in nature; arising as and when circumstances dictate. For example, at a basic level, the state (as a labour market actor) may be forced to close various regulatory ‘loopholes’ and grey zones exploited by employers such as the misclassification of platform workers as ‘independent contractors’, particularly where such practices threaten the tax base (e.g. Adler, 2021; Jaehrling and Kalina, 2020). Existing rules such as minimum wages and basic employment rights and social protection may be extended to those in non-standard forms of employment (Poblete, 2020), or governments may create wholly new forms of ‘dependent’ self-employment contracts that seek to balance flexibility and security (López-Andreu, 2020).

However, state experimentation in response to disruption is often slow and partial, and social actors such as trade unions, NGOs and grassroots community activists are often better placed to articulate the specific challenges facing workers and citizens brought about by economic restructuring. This may be through new repertoires of contention that transcend traditional trade union territories and incorporate community organising (e.g. Atzeni, 2020; Milkman, 2019), social and political campaigning (e.g. Rhomberg, 2018) and coalition building (e.g. Tattersall, 2018). Similarly, smaller ‘independent’ unions such as the Industrial Workers of the World (IWW) and Independent Workers of Great Britain (IWGB) are seen as less bureaucratic and ‘top-down’ than traditional unions, who can quickly mobilise both structural and discursive power resources to challenge managerialism and exploitation (Bertolini and Dukes, 2021; Però, 2020; Smith, 2022). The emergence of ‘worker centres’ across the US over the last 20 years or so is a direct response to widespread non-compliance with existing laws and regulations around contract types, minimum wages, social security contributions, and health and safety standards, particularly in areas where migrant workers are concentrated and unions are weak (e.g. Fine and Bartley, 2019). In the UK there is a small network of unemployed worker centres (UWCs), mostly in the Midlands and north of England, who fulfil some of the same regulatory functions (albeit in a less expansive and coordinated way) (Griffin, 2023). Legal advice and advocacy services have survived in some deprived communities in the UK, but they typically lack the resources to organise and mobilise hard-to-reach groups (Mustchin et al., 2023).

### Hybridisation

Hybridisation reflects the combination of old or institutionally embedded practices and new processes and activities that are in some way different and developed by actors under pressure from disruptive forces (such as organisational fragmentation and regulatory reconfiguration). Over time actors learn from successes and failures and begin to seek out the necessary opportunities, resources and allies, to rewrite institutional rules and create new shared understandings of what constitute fair or decent minimum standards (Murray et al., 2020). For example, even so-called ‘business’ or ‘servicing’ unions have incorporated dynamic organising strategies aimed at greenfield industries and under-represented groups in precarious jobs (Keizer et al., 2023). Many unions have also embraced the use of digital technologies and social media in order to engage with younger and more isolated workers who do not necessarily have a fixed workplace (Hodder and Houghton, 2020). Similarly, the incorporation of various labour clauses into public procurement exercises can be viewed as a complement to established union demands for secure work and fair pay as well as the historical (but by no means unequivocal) position of the state as a good employer (Grimshaw et al., 2016). Recent studies have also explored coalitions of trade unions, CSOs and (sometimes looser) social movements, whose combined power resources may exceed that of any one individual actor (e.g. Frangi et al., 2020; Tattersall, 2018). Experimental practices may also become increasingly embedded over time as they become normalised and legitimised, further blurring the boundaries between old and new. For example, the Living wage/Fight for $15 campaign that started in the US in the late 1990s was initially driven by networks of local community and faith activists, but the campaign draws heavily on the institutional resources of trade unions and NGOs who have the leverage and legitimacy to put pressure on big employers and state actors to increase wages (Rhomberg, 2018).

Where social actors are weaker and more fragmented, their responses may be uncoordinated and incremental as the true nature and extent of disruptions are revealed, and the power resources required to respond effectively are mobilised. Indeed, while hybridisation suggests a purposeful combination of old and new practices, the concept of ‘bricolage’ reflects the pragmatic creation of something innovative using available materials or resources and adapting old strategies to new contexts. For example, migrant workers may find themselves channelled into the lowest paid and most precarious jobs (such as semi-formal gig work and day labour), but recent studies also show how as ‘bricoleurs’ they can make use of their dense social networks (on- and offline), and their lived experience of the peculiarities of specific work, welfare and migration systems to both advise and organise others within the international diaspora (Castellani and Roca, 2022).

## Local disruption and local experimentation

Given that the specific manifestations of labour market exploitation and inequality are increasingly visible within local rather than national capitalisms (Gough, 2014), it is also often (but not always) within local and urban contexts where processes of experimentation and hybridisation play out. For example, cities are often broadly ‘progressive spaces’ where egalitarian policies may mirror the political leanings and social justice concerns of metropolitan citizens (e.g. Joy and Vogel, 2021). In addition, local politicians (and city mayors in particular) are seen as pragmatic and open to new ideas, and through their extensive local networks are able to ‘get things done’ (Ayres, 2022). In addition to these top-down forms of experimentation, there are bottom-up pressures for change that arise from the particular organising and mobilising capacities and networks of individual workers, trade unions, social movements and communities (Castellani and Roca, 2022; Frangi et al., 2020). For example, in the US there is a burgeoning literature on state and city level regulatory innovations around labour market standards such as local minimum wage and living wage laws and specific protections against wage theft (Jacobs et al., 2021). These responses link in with a more general trend towards the replacement of collectively agreed standards by legal minima, although these are often only a partial substitute, and suffer widespread problems of non-compliance (Fine and Bartley, 2019). There are also particular concentrations of industries and employers within cities that may be responsive to the development of progressive employment norms, or may alternatively push back against, or seek to exit new regulatory and normative structures (Adler, 2021).

In trying to regulate local capitalisms, state actors are likely to draw on soft and convening power to build partnerships with other stakeholders rather than relying on simple coercion and the enforcement of rules. One prominent initiative within a UK context is Scotland’s Fair Work Convention, sponsored by the Scottish National Party, which encompasses worker voice and collective bargaining, as well as security, respect, opportunity and fulfilment, but largely relies on the business case of ‘mutual gains’ partnerships rather than hard legal regulation (Cunningham et al., 2022). In the absence of significant law-making powers at local level, the notion of ‘legitimacy’ provides an alternative potential (albeit softer) constraint on the behaviour of the parties within the employment relationship (Doellgast et al., 2021). For example, businesses, however powerful and globally dispersed, still rely on the support of multiple stakeholders, including customers, employees and regulators, and are vulnerable to negative responses to behaviours that are considered to be ‘illegitimate’. Indeed, experimentation and hybridisation may involve the development of new cognitive frames and shared understandings about the legitimacy and fairness of particular organisational and institutional behaviours and may result in new ways of thinking and acting in particular contexts (Murray et al., 2020). Considering the interplay of institutions and actors provides a framework for understanding how support for specific practices is maintained over time, or where embedded practices lose their legitimacy because of waning stakeholder consensus and changes in the economic and social context (e.g. Chaudhry and Rubery, 2019).

The review of relevant literature generates three main questions that guided our research. The first question is: what evidence is there of innovation and experimentation within the UK in response to labour market disruptions? The second is how do such experiments interact with embedded processes and activities to produce hybrid forms of action? The third question is what is the role of legitimacy in contributing to the elaboration and maintenance of labour market norms at the local level?

## Research context and methods

The research follows a qualitative case study methodology (Yin, 2017) that seeks to understand the process and dynamics of experimentation and re-regulation within a specific city region, positioned within a wider UK context of neoliberal economic policy making and welfare state retrenchment, sustained organisational and labour market fragmentation, and the steady decline of collective worker representation.

Greater Manchester (GM) was selected as a ‘critical case’ as it has faced significant economic and social disruptions over the last 40 years or so but has seen some important localised regulatory experiments in recent years, coupled with significant mobilisations of social actors (both before and after Covid-19). While focusing on one specific English city region may potentially limit the generalisability of the findings to other geographical areas, it also allows us to better elaborate the local economic, institutional and political context within which these actions and strategies have emerged. This in turn may provide some important reference points for other post-industrial cities and regions around the world. Our core proposition is that the history of trade unionism and left-wing political control within GM makes it an important space for innovation in respect of re-regulating labour markets and developing socially inclusive approaches to work and employment. Layered on top of these dynamics are the recent tentative moves towards devolution, as Greater Manchester was one of the first city regions to receive localised powers and resources to coordinate public services and transportation. At the same time, high levels of unemployment in some parts of the city region, coupled with persistent and extensive problems of low paid and precarious work mean that progressive approaches within GM may be undermined by deep rooted structural imbalances in national (and international) economic systems. The case study of GM provides some important clues as to how the tensions between an increasingly globalised capitalist system of economic development and the recent turn towards local progressive politics may be resolved (Gough, 2014).

The data reported in this article are part of a larger international programme of research on work and employment that has so far amassed more than 100 interviews. The empirical findings analysed here draw on 40 in-depth, face-to-face, semi-structured qualitative interviews with key stakeholders from across the GM city region over two years (2021–2023). The research adopted a purposive sampling strategy that sought to triangulate the viewpoints of diverse stakeholders about processes of change within GM, and to reveal how local actors both shape and respond to labour market disruptions, and efforts to re-regulate work and employment. The sample of interviewees included multiple local and city region government officers at different levels of seniority; a range of trade union representatives from the North West Trades Union Congress (NWTUC), UNISON, Unite and the GMB; business organisations, such as the GM Chambers of Commerce; professional HR and employment relations organisations, such as the Advisory Conciliation and Arbitration Service (ACAS) and the Chartered Institute for Personnel and Development (CIPD); along with civil society organisations, such as Citizens Advice (CA), the Greater Manchester Law Centre and Greater Manchester Poverty Action (GMPA). The sample also included individual trade unionists and activists involved in specific local campaigns.

Interviews lasted between 45 and 90 minutes and were audio recorded and transcribed verbatim. A systematic thematic analysis was undertaken, aligned to the research objectives and key issues identified in the literature, such as the perceptions of specific disruptions and transformations in the labour market, the process of experimentation and innovation at local level, the interactions between new and embedded practices, and the way in which rules and norms were articulated and legitimised by different actors. This was complemented with extensive desk research that examined existing local policy documents (in particular the Good Employment Charter, the GM Challenger Programme and the Living Wage city region action plan), newspaper reports and recently published economic and labour market reports in order to identify key trends and emerging policy issues around work and employment.

## The varied responses to disruption in Greater Manchester

Our findings reveal that a combination of long- and short-term disruptions incentivised established social actors to consolidate and defend existing institutional agreements while also creating spaces for experimentation with new approaches that sought to promote good employment and responsible business. Importantly, these experiments were a combination of top-down political ‘projects’ and bottom-up organising and activism. There were also examples of hybrid approaches that brought together trade unions and community organisers around specific ‘single-issue’ campaigns.

### Embedded practices: Threats and opportunities

For social actors within GM, the ‘disruption’ brought about by labour market fragmentation was twofold. Firstly, a long-run pattern of economic restructuring since the late 1980s saw the rapid decline of manufacturing and heavy industry and contributed to high and persistent unemployment in parts of the region. These relatively stable (but not always well paid) jobs were replaced by service sector jobs that were often precarious, part-time and low paid, which contributed to a growing problem of low productivity and in-work poverty. The second ‘disruption’ was the perceived failure of national government to successfully address regional disparities in economic and social outcomes. These issues were exacerbated by the 2008–2009 global financial crisis and subsequent top-down public sector cutbacks that compounded economic and social exclusion across the city region. As a result, Greater Manchester is consistently ranked among the worst in the UK for incomes, living standards and productivity and, despite the central government devolution deal of 2016, relatively few powers and resources have been transferred to the local level, save for some limited responsibility for transport and health (Pickett et al., 2020).

Despite these challenges, trade union membership has held up well in core industries such as parts of the public sector, public utilities and transport, and larger trade unions such as UNISON, Unite and the GMB have sought to defend hard-won terms and conditions against the general downward pressure of austerity after 2010, as well as the more acute disruptions brought about by Covid-19 (although not always wholly successfully). Significant public demonstrations against austerity, partly coordinated by the trade unions, have taken place within Manchester since 2010, and national strikes of public sector workers in 2011 and 2014 were well observed within the city region. GM is also home to several universities which have seen significant national and local strike action over pay and pensions led by the University and College Union (UCU) since 2018.

Trade unions and the TUC within GM have a history of partnership working with left-wing politicians across the region, which gave them input into local economic policy making, although arrival of a directly elected mayor after 2017 threatened to disrupt these relationships (see next section). The public sector trade union UNISON has leveraged its considerable local institutional power resources to develop broader campaigns around investment in public services and the need to better protect frontline essential workers from both physical and economic risks. The Covid-19 pandemic exposed significant challenges around care workers’ lack of access to personal protective equipment (PPE), as well as the limited entitlements to occupational sick pay (owing to low/variable hours and earnings). The ‘Care vs. Covid’ campaign aimed to secure full pay for care workers who had to isolate due to Covid-19 and in turn provided a platform for in-fill recruitment among the social care workforce across both the public and private sectors. The social care sector has become the focus of trade union efforts to extend decent minimum rates of pay to outsourced workers and since 2019, across GM an agreement has been reached to allocate a minimum of 20% of external contract marks to ‘non-commercial’ considerations, such as hiring local workers, providing training and development opportunities, and guaranteeing ‘good’ employment conditions such as the living wage and secure jobs. These standards have been applied to larger contracts across the GM city region such as waste disposal and major construction projects, and individual local authorities can choose to augment these standards through their own procurement processes for outsourced services such as social care, catering and cleaning. This was seen as important for the trade unions, who argued that larger public contracts were a platform for raising standards along increasingly long and complex supply chains:

. . . where you have got those big contracts and you have got the big spending power, then you’ve got more scope of what you can do. (TUC representative)

In additional to ‘traditional’ trade union activities such as political lobbying and casework, union branches across GM have been involved in a number of workplace disputes since Covid-19. For example, in early 2021 a significant dispute erupted between bus drivers (represented by the trade union Unite) and their private sector employer (GO North West). The dispute centred around management’s attempted use of ‘fire and rehire’ provisions to force through changes to working practices that would extend working hours and reduce sick pay for those with fewer than five years’ service. Workers were particularly aggrieved that after working throughout the pandemic to transport other essential workers around the city (often with inadequate health and safety measures) management had sought to renege on hard-won provisions around shift patterns and working time. Unite sought to reach out to and build solidarity with other unions and local grassroots campaigners through virtual events and (socially distanced) public protests and demonstrations, but fundamentally, high membership density within the privatised bus network built up over a number of years provided a strong platform for collective action. The vote for strike action was near unanimous and resulted in a near complete suspension of bus services in some parts of the city over a period of 82 days as picket lines effectively blockaded key depots:

. . . because we’d got at that time 99% union membership nobody’s walking over the line. (Unite local organiser)

National trade union negotiators worked closely with local union organisers and in the end while Unite members agreed some concessions on efficiency savings, the main proposal to effectively downgrade pay and conditions through the controversial use of ‘fire and rehire’ procedures was withdrawn across the GO group^1^ after nearly 11 weeks of sustained industrial action. A subsequent strike of Unite workers at another bus depot achieved a 9% pay rise without concessions on previously negotiated terms and conditions.

While indefinite strike action is not unheard of in the UK, it is not common owing to the increasingly tight restrictions on industrial action since the 2016 Trade Union legislation that requires both a majority vote and at least a 50% turnout of eligible members in order for strike action to be lawful. The requirement to renew strike mandates every six months also creates additional barriers to continuous action. Nevertheless, a number of other long-running disputes following the Covid-19 pandemic highlighted the importance of embedded power resources within organised workplaces. An all-out strike at a pallet supplier during 2021/2022 over pay and conditions lasted nearly five months and drew on significant structural and associational power resources in order to force management to increase their original offer of just 1.8% to 9%, along with three extra days of annual leave, and a £1,000 lump sum. While many workers had been on strike before, they had not participated in sustained action over such a long period and the picket lines were well attended for the full five months, which in turn drew support from other unions and the general public. Manchester Trades Union Council (MTUC) also leveraged its significant local networks and connections to raise additional strike funds in order to support the workers.

### Experimentation: Top-down and bottom-up responses to disruption

The complex and ongoing nature of disruption within Greater Manchester created the impetus for experimentation, which involved a wide range of social actors (both new and old) and multiple power resources. Importantly, the experiments observed were a combination of top-down (e.g. led by established and relatively powerful social actors aligned with higher level political and policy agendas) and bottom-up (e.g. more spontaneous and driven by the immediate real-world challenges faced by specific groups of workers and citizens).

The Greater Manchester ‘Good Employment Charter’ is an important example of a local top-down state-led experiment to raise living standards in the region through better pay and employment conditions, linked with improved skills and productivity. This experiment not only led to the building of new coalitions of state actors, employers, trade unions and civil society, but also provided a template for similar soft-regulatory projects in other cities and regions across the UK. Political actors within GM had long been calling for greater devolved resources to address policy issues locally and in 2016 the first city region devolution deal was agreed with GM which transferred some limited decision-making powers in return for the requirement to create the role of a directly elected mayor (Fahnbulleh et al., 2022). In contrast with the US model of strong city mayors who determine taxation, investment and regulation issues at local level, the emergent model of city region mayors in the UK draws more heavily on soft power and the ability to coordinate actors around a common purpose:

Actually, the mayor in Greater Manchester has very few powers it turns out, he or she can have a lot of convening power . . . and, particularly when they are a high-profile media figure with a national profile who is brilliant at communicating but the actual formal powers of the mayor to decide [on issues] without asking anybody else’s permission [they are] very little. (City region government official)

As part of his manifesto in 2017, Labour Party politician Andy Burnham pledged to create a ‘Good Employment Charter’ in order to improve job quality across the city region. The charter was developed in discussion with local employers, NGOs and trade unions, and included seven key dimensions: flexible work; a real living wage; workplace engagement and voice; excellent recruitment practices and progression; excellent people management; and a productive and healthy workplace. But rather than the Mayor simply imposing these standards on businesses, the aim was to create a legitimate ‘movement’ of diverse local actors (including employers, trade unions and civil society) that positively engaged with the norms of ‘responsible business’ and ‘good employment’. By the end of 2022 there were 59 companies registered as full members of the charter (and fulfilled all seven dimensions) and 421 as supporters (who were working towards membership). However, these were mostly larger public and private sector firms with a local base (and not necessarily headquartered in the area) and relatively few were small employers in low margin sectors such as care, retail and hospitality where issues of poor wages and insecure work are typically concentrated.

More broadly, while the Mayor successfully established a normative frame of ‘good employment’ and ‘responsible business’, the question remains as to what counts as ‘bad’ employment or ‘irresponsible business’ and what should be done to tackle these entrenched problems. Local government officers involved in the Good Employment Charter recognised that soft regulatory measures would not have much impact on the margins of the labour market, where a more aggressive and intelligence led approach was required. The GM Challenger Programme is another top-down experiment^2^ built around a multi-agency ‘modern slavery’ unit established in 2015 consisting of specialist police officers, data analysis and support staff. Modern slavery, however, is something of a catch-all, and ranges from wholly informal and illegal activity where workers are trafficked and controlled by organised gangs, through to cash-based businesses such as takeaways and hand car washes that often do not comply with minimum wage or health and safety regulations (see also Clark and Colling, 2019). And while the Challenger Programme was well respected in the local area, in the absence of significant devolved regulatory powers the focus was largely on investigating the most egregious cases of exploitation and non-compliance (Davies, 2019).

For more ‘routine’ cases of non-compliance, civil society actors such as Citizens Advice have a small number of employment advisors who can provide individual casework and support for those pursuing an employment tribunal claim and has also undertaken campaigning activities around welfare reform and issues of non-compliance with the minimum wage. However, austerity pressures have led to the retreat of many state and civil society services within deprived communities, which in turn has created a space for further bottom-up experimentation. For example, the GM Law Centre was established in 2017 by a local network of trade unionists, lawyers and community activists in response to the closure of a prominent local legal advice centre in 2014, and the national withdrawal of legal aid for employment issues in 2013. Legal advice centres have emerged in a number of UK cities over the last decade (partly building on the legacy of Unemployed Worker Centres set up in the 1980s) and while they have not yet achieved the regulatory influence of worker centres in the US, they are engaged in a wide range of individual advocacy and collective capacity building (Griffin, 2023). For example, the GM Law Centre actively promotes trade union membership and has collaborated with unions and civil society organisations on specific campaigns, such as employment rights, access to justice and preventing evictions. This broader campaigning has become particularly important as with limited resources the centre was struggling to keep up with the demand for pro-bono legal advice and support around individual workplace issues:

. . . since our employment lawyer started in January last year [they have] advised 327 people in Greater Manchester on employment matters. (GM Law Centre director)

As another potential site of bottom-up experimentation, grassroots unionism and worker ‘self-organisation’ have developed somewhat sporadically across GM and have often been centred around specific sectors and groups of workers such as hospitality, and platform food delivery riders. Smaller ‘indie’ unions such as the Industrial Workers of the World (IWW), the Independent Workers of Great Britain (IWGB) and the App Drivers and Couriers Union (ADCU) have all organised strike action, ‘coordinated logoffs’ and public demonstrations among platform workers (mostly Uber and Deliveroo) and sought to build organising cultures among local networks of migrants (mostly from South Asia, Southern Europe and parts of Africa). However, the resource intensive nature of sustained organising, particularly within a highly dynamic workforce spread across various parts of GM, meant that early wins were difficult to sustain when compared with larger cities such as London where trade union resources were concentrated, and greater political and media exposure could be achieved. In this context, some drivers and riders have since joined larger trade unions such as the GMB who have followed a more traditional workplace recruitment model where full-time officers emphasise the individual benefits of membership such as support and legal advice during grievance and de-activation procedures. Organisers also explicitly reference the recently signed recognition deals with both Uber and Deliveroo and the 12% pay rise that Uber drivers received as a result of the Supreme Court ruling on limb-b worker status that entitles workers to pro-rata holiday pay. Any successful outcomes of this individual servicing approach were then used to recruit new members through word of mouth:

. . . you’ll get . . . another two or three members joining online and probably never even see them. (GMB regional organiser)

### Hybridisation: Learning from successes and failures

The final dimension of re-regulation in GM is the emergence of hybrid approaches that brought together traditional and new actors around single-issue campaigns, namely: the GM living wage campaign; and a joint union–community campaign to maintain state funded nursery provision.

The living wage campaign is one of the most well-known and arguably successful social justice campaigns of the last 30 years, which despite its global spread often remains highly attuned to local economic and political contexts. The UK living wage campaign emerged in the late 1990s in the east end of London and around Canary Wharf and targeted blue-chip firms before moving on to public institutions such as the Greater London Assembly and hospitals in London that outsourced many low paying jobs. The UK campaign is relatively centralised when compared to the city-level campaigns in the US and has typically relied on the campaigning expertise and resources of larger civil society organisations, such as Citizens UK and the Living Wage Foundation, to establish a consistent message and methodology for calculating living wage rates. At the same time, local campaigns such as the GM living wage campaign have been given impetus and energy by the same dynamic coalitions of labour, faith and community groups in US cities that were able to mobilise around issues of inequality and insecurity at work.

The trade unions within GM have long argued that the statutory minimum wage should move towards the real living wage in order to reflect the actual cost of living and have used their collective bargaining agreements within the public sector to ensure that the lowest paid workers are paid at least the living wage. But the main driving force behind private sector employers adopting the real living wage is the GM living wage campaign established in 2013. The GM campaign is one of the most well recognised outside of London, and the involvement of Greater Manchester Poverty Action (GMPA) provides a high degree of legitimacy when discussing issues of inequality and in-work poverty. One campaigner recognised that the success of the campaign was partly as a result of making a strong business case to ‘enlightened employers’ (particularly after Covid-19 when labour shortages intensified), but also lamented the move away from recruiting activists and leveraging direct-action tactics:

. . . I don’t think we do enough [direct action] it’s good for publicity to get 20 people stood outside an employer with placards . . . and the local press will always pick stuff like that up . . . but I’m not pushing many tanks around: I’m not deploying many troops. (GM living wage campaigner)

Some of these tensions were also evident in the incorporation of the living wage into the Mayor’s Good Employment Charter, and the development of the GM living wage city region ‘action group’. The adoption of the real living wage (RLW) as a ‘red-line’ minimum standard for charter membership was a reflection of both trade union and campaign pressure, as well as the broader legitimacy of the RLW as a benchmark of decent work within GM. However, it also meant that the living wage is at risk of being ‘captured’ by local political and business elites who have partly re-framed it as an issue of productivity and economic growth as opposed to one of social justice and worker organising. The RLW action group consists of business and civil society leaders who utilise their networks and influence to promote the living wage across GM, with a particular focus on locally based SMEs and low paying industries such as health and social care. As with the charter, the scaling up of the living wage campaign points to a move from organisational to institutional experimentation, but the trade-off is increased bureaucracy and a widening gap between leaders and activists (Murray et al., 2020).

A second important hybrid experiment that occurred in Greater Manchester was a joint trade union/community campaign to stop the closure of five nurseries (for pre-school children) in Salford. The nurseries were somewhat unusual for the UK in that they were still owned and managed by the local authority, and workers were highly unionised and still covered by the local government sectoral collective agreement. This meant higher wages and access to a pension scheme (£10+ per hour compared with the minimum wage of £7.83 at the time), lower staff turnover and high-quality provision for children, particularly those with special educational needs.

In early 2018, Salford local authority announced plans to put the nurseries up for sale and to transfer staff and services to a third-party provider or alternatively they would close and staff would be redeployed elsewhere in the council. The council claimed that restructuring was a result of long-term reductions in grants from central government and attempted to enlist parents, workers and UNISON (the recognised trade union) representatives to lobby national politicians in order to release more funds in order to safeguard the nurseries. At the first public consultation meeting parents and workers filled the main room as well as the lobby and entry area and made it clear to politicians and senior officers that they would not simply accept the nursery closures without a fight. But rather than UNISON simply leading a conventional campaign to defend public services and collectively agreed pay and conditions, they quickly formed a coalition with parents and community actors who were able to mobilise broader support for the quality of education provided within the nurseries and the crucial role they played in supporting deprived communities:

. . . we left that meeting and we just shouted it from the rooftops this is what’s going to happen, these five nurseries are going to close . . . we got in touch with staff who wanted to defend their nurseries and with their help and their involvement, we got the message out to parents via social media. (UNISON organiser)

The campaigners successfully hosted large public rallies and petitioned local politicians to pause the consultation, and after a period of reconciliation with senior figures within the council they were able to agree a set of minimum employment and service quality standards that any external provider would have to comply with (which effectively kept the nurseries in public ownership as no external provider could be found that met these standards). The joint campaign between trade unions, workers and service users was replicated in other cities around the UK to defend state-maintained nurseries (but with little success), and also became a template for other local campaigns within GM when public services have been threatened with closure (to greater success). This is an important legacy of a single-issue campaign whereby a range of actors combined traditional and new strategies in response to the ongoing disruption of public sector retrenchment.

## Conclusion

Through an extended critical case study of the Greater Manchester (GM) city region in the UK, this article explored processes of regulatory experimentation and innovation at local level, and how new practices interact with, add to, and sometimes challenge embedded ways of thinking and acting (Murray et al., 2020). Through qualitative interviews we mapped the increasingly diverse range of actors involved in processes of labour market re-regulation within GM, and how they sought to develop new solutions to perceived economic and social disruptions. In the case of GM, actors are responding to the long-term disruptions of neoliberal policy making since the 1980s, the steady decline of manufacturing and heavy industry, and the rise of in-work poverty driven by a rapid expansion in low paid and precarious service sector jobs. Layered on top of these cumulative disruptions are the socio-economic damage caused by Brexit and the Covid-19 pandemic, a sustained period of fiscal austerity since 2010, and the growing disillusionment with the national government’s rhetorical commitment to city region devolution and ‘levelling up’ that has yet to transfer any meaningful resources or powers to local level.

Mapping out the specific responses to disruption in GM, as well as the actors involved (Table 1), shows how processes of de-regulation and marketisation pose challenges for notions of cooperative ‘partnership’ working between social actors within GM, and in some cases short-term ruptures in workplace employment relations (particularly after Covid-19) have provoked significant resistance and mobilisations by trade union members. More broadly, the increasingly permissive regulatory context of the UK has also created the impetus for significant local experimentation, which as this article has shown are both top-down and bottom-up in nature. Finally, there are important examples of hybridisation and bricolage that have proven to be relatively successful in relation to single-issue campaigns such as the living wage and publicly funded nurseries.

**Table 1.** Institutional responses to disruption in Greater Manchester.

	Nature of response	Actors involved	Strategies and tactics
***Embedded practices***	Collective bargaining, political lobbying, workplace organising, individual casework	Trade unions (UNISON, Unite, GMB)	Consolidating power resources in key industries, successful use of continuous strike action, coordinated fund raising and media work
***Experimentation***	***Top-down*** Soft regulation (Good Employment Charter, social value procurement) Enforcement of modern slavery law ***Bottom-up*** Worker advocacy and representation, organising and capacity building	State (mayor’s office and civil servants), larger employers, trade unions and civil society leaders Civil society organisations, ‘indie’ trade unions	Using convening and legitimacy power to build support for good employment agenda Using grounded knowledge and local networks to raise awareness and protect vulnerable workers
***Hybridisation***	Living wage and anti-poverty campaigns, campaign to defend public services	Civil society, communities, service users, trade unions	Building new partnerships and coalitions, mixture of conflict and cooperation, political lobbying

Taken together, our case study data allow us to make two substantive contributions. Firstly, our findings suggest that the focus on innovation and experimentation should not mean we overlook the important role of established actors who draw on ‘traditional’ repertoires of action and frames of understanding in order to defend hard-won employment standards. It is the institutionalised nature of the relationships between social partners with GM that has created a space for the discussion of broad policy agendas around decent work and inclusive growth (Johnson et al., 2023; Lupton et al., 2019). Similarly, the power resources held by larger trade unions within core industries provide a platform for the effective articulation of key grievances and afford a degree of leverage against opportunistic employers who have sought to use the disruption of Covid-19 to erode core standards (Herman et al., 2021). Our findings reveal complex patterns of institutional ‘layering’, rather than more abrupt processes of replacement and breakdown that may follow from institutional ruptures and change (Murray et al., 2020; Thelen, 2009). For example, while continuous strike action represents something of a departure for local industrial disputes in GM, the effectiveness of the ‘strike weapon’ is a reflection of sustained workplace organising by various trade unions such as UNISON, Unite and the UCU, and interunion solidarity fostered by the North West Trades Union Congress (NWTUC) and Manchester Trades Union Council (MTUC). Similarly, larger national unions such as the GMB have moved into the spaces left by smaller activist unions such as the IWW and IWGB, who have struggled to expand outside of London where their activist resources and networks are concentrated (Però, 2020; Smith, 2022). It is the fluid combination of embedded power resources, suffused with the ideas and skills of workers, activists and citizens, that has been a major source of success in single-issue campaigns such as the living wage and maintaining publicly funded nurseries. These hybrid approaches suggest that rather than competition between ‘old’ and ‘new’ actors for resources and representative claims, there are increasing opportunities, and incentives, for a plurality of social partners to form alliances and coalitions around broader social justice concerns (e.g. Tattersall, 2018). The key issue here is the extent to which these localised campaigns and coalitions can be scaled up and replicated across geographical regions to deliver gains for a wide range of workers and citizens.

This attention to context and scale underpins our second contribution. Our findings suggest that, as in the US, there are important experimentations at local level that partly counteract the permissive regulatory context that has normalised non-compliance with basic standards such as health and safety laws and minimum wages (Fine and Bartley, 2019; Jacobs et al., 2021; Judge and Slaughter, 2023). In England, however, local experiments (whether top-down or bottom-up) are typically framed by softer notions of shared ‘norms’ and ‘aspirations’ rather than binding rules, which actors are incentivised to comply with out of concerns for legitimacy in the eyes of key stakeholders rather than the coercive power of the state (MacKenzie and Martínez Lucio, 2014). The purchasing influence of local ‘anchor institutions’ is increasingly being used to set decent minimum standards within local supply chains (Lupton et al., 2019), and this approach has been successful in the devolved governments of Wales and Scotland (e.g. Cunningham et al., 2022). However, English city region mayors do not have the same devolved powers over labour market and public service issues (aside from parts of transportation), and generally have to govern by consensus rather than by diktat (Sandford, 2020). As the legitimacy and reach of state institutions shrink further, networks of activists and grassroots organisers within communities have become increasingly central to the efforts to re-regulate labour markets ‘from below’ (Fine and Bartley, 2019; Lesniewski and Canon, 2016). The challenge is that civil society and community actors in the UK have only recently begun to experiment with the campaigning and organising activities pioneered by worker centres in the US (Mustchin et al., 2023). Our findings show that a critical mass of social actors rooted in the local context may be necessary for the development of collective capabilities that advance a long-term progressive agenda (beyond just short-term and reactive experiments; Murray et al., 2020; Rhomberg, 2018).

Taken together, our findings suggest that the distinctions between embedded, new and hybrid practices are somewhat artificial; what are arguably of more interest, and may carry more explanatory power, are the interactions between organisations and actors that result in the ‘layering up’ of multiple conventions, norms and rules that help shape individual and collective behaviours (Thelen, 2009). On the one hand, new rules (and associated patterns of behaviour) can emerge from processes of bricolage and the seemingly uncoordinated actions of various individuals rather than a unified collective choice at a point in time, or predictable political processes (Castellani and Roca, 2022). On the other hand, our findings reveal evidence of both top-down and bottom-up experimentations taking place within a relatively stable and broadly progressive political context, albeit one that has come under sustained disruptive pressure from external forces of economic restructuring and centralised welfare cutbacks (Gough, 2014; Yates and Clark, 2021). While local experiments are important and necessary responses to ever widening fault lines in the labour market, they do not operate in a vacuum, and draw their power and legitimacy from embedded organisational and institutional contingencies such as progressive political traditions, coordinated and visible trade unions, active civil society, and well-established social justice campaigns. It is only by carefully mapping these contextual factors, and how they shape shared norms around decent minimum standards at work, that we can understand the opportunities, and limitations, of sub-national experiments to re-regulate labour markets.

## Notes

1. Officers within Unite shared the details of the dispute with trade union colleagues in Sweden (where fire and re-hire is illegal) as GO was bidding for other transportation contracts at the time.

2. Following the 2015 Modern Slavery Act aimed at tackling human trafficking (legislation.gov.uk)
